# Limb salvage using Pounce LP mechanical thrombectomy system after failed open and percutaneous thromboembolectomy

**DOI:** 10.1016/j.jvscit.2025.101935

**Published:** 2025-07-29

**Authors:** Hassan Chamseddine, Mouhammad Halabi, Alexander Shepard, Loay Kabbani, Kevin Onofrey

**Affiliations:** Division of Vascular Surgery, Department of Surgery, Henry Ford Hospital, Detroit, MI

**Keywords:** Acute limb ischemia, Mechanical thrombectomy, Pounce LP thrombectomy system

## Abstract

Mechanical thrombectomy devices have limited efficacy in organized subacute and chronic thrombi and face challenges in small-caliber distal vessels. The Pounce LP Thrombectomy System (Surmodics) is a fully percutaneous, purely mechanical thrombectomy device designed to overcome these limitations. We report the case of an 83-year-old female presenting to an out-of-state hospital with acute limb ischemia secondary to a right external iliac artery occlusion. Despite percutaneous mechanical thrombectomy with an aspiration thrombectomy device and subsequent open Fogarty balloon thromboembolectomy for distal embolization, her ischemia worsened, and a below-knee amputation was recommended. Upon transfer to our institution, she had sensorimotor deficits, distal ischemic changes, and occlusions of the anterior tibial, posterior tibial, and peroneal arteries at the level of the midcalf. Mechanical thrombectomy using the Pounce LP device successfully removed organized thrombus and restored multiphasic inline flow to the peroneal artery and its communicating branch, which subsequently fed into the distal posterior tibial artery and the plantar vessels. The patient ultimately underwent a successful transmetatarsal amputation, achieving limb salvage.

Acute limb ischemia (ALI) is a sudden and significant decrease in limb perfusion that threatens limb viability and can lead to both amputation and death.^1^^,^^2^ It is considered a highly morbid vascular emergency, with limb amputation rates as high as 40% and mortality reaching 20%, even with adequate treatment for patients presenting with more advanced ischemia.^3^^,^^4^ Traditionally, ALI in the United States has been treated with open surgery.^5^ More recently introduced endovascular interventions are less invasive and have a lower procedure-associated morbidity.^6^

Although catheter-directed thrombolysis, mechanical thrombectomy, and aspiration thrombectomy are all effective in removing fresh acute thrombus, their efficacy diminishes with more organized subacute and chronic thrombus. Additionally, commercially available thrombectomy devices become less effective and more challenging to use in small-caliber distal and pedal vessels. Introduced in 2024, the Pounce LP Thrombectomy System (Surmodics) is a fully percutaneous, purely mechanical thrombectomy device specifically designed to address these limitations. It has been reported to safely and effectively remove both acute and subacute thrombus in peripheral vessels measuring 2 to 4 mm, making it particularly well-suited for tibial and pedal artery occlusions.^7^

We report a case of successful limb salvage after ALI using the Pounce LP Thrombectomy System following multiple failed open and percutaneous thrombectomy attempts. The patient has provided consent for publication of this case.

## Case report

An 83-year-old female patient with a history of hypertension, hyperlipidemia, atrial fibrillation, polycythemia vera, and chronic kidney disease presented initially to an out-of-state hospital with a 48-hour history of acute onset of right foot pain, paresthesia, and cyanosis. Computed tomography angiography (CTA) revealed a right external iliac artery occlusion with distal embolization for which she underwent percutaneous mechanical thrombectomy using an aspiration thrombectomy device and achieving postprocedural flow in the posterior tibial (PT) artery. Worsening of the tingling sensation in the foot at rest 48 hours after the initial intervention prompted a repeat CTA that demonstrated reocclusion of the right external iliac artery with distal embolization to the tibial arteries, despite therapeutic anticoagulation with apixaban. The patient had no palpable pulse or Doppler flow in the PT and dorsalis pedis (DP) arteries and subsequently underwent open Fogarty balloon thromboembolectomy via right femoral and popliteal artery exposures. Although Doppler examination reportedly documented restored monophasic flow in the PT artery at the ankle, ischemic changes and pain progressed in the right forefoot. The patient was told there were no further therapeutic options available, and a below-knee amputation was advised.

The patient requested a second opinion and was subsequently transferred to our institution on the fifth postoperative day after her initial symptoms. On presentation to our institution, physical examination revealed sensorimotor deficits in the right forefoot, distal ischemic changes (Supplementary Fig, online only), a faint PT artery Doppler signal, and an absent DP artery signal. The right mid and proximal foot retained adequate sensorimotor function. Intravenous heparin was initiated, and CTA revealed patent right iliac, femoral, and popliteal arteries with occlusions of the anterior tibial, PT, and peroneal arteries at the level of the midcalf.

**Fig 1:**
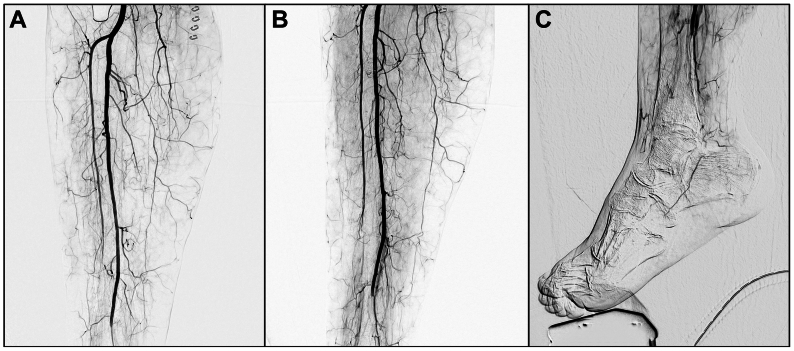
Intraoperative angiography showing occlusions of the anterior tibial, posterior tibial (PT), and peroneal arteries **(A** and **B)** and absent distal flow to the right foot **(C)**.

**Fig 2:**
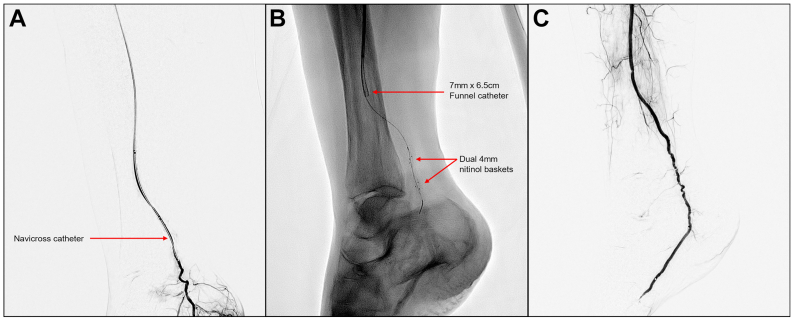
**(A)** Traversing the distal posterior tibial (PT) artery via a Navicross catheter; **(B)** dual 4-mm diameter self-expanding nitinol baskets in the distal PT artery; and **(C)** completion angiography showing arterial flow to the midfoot through the medial plantar artery.

The patient was taken to the operating room for mechanical thrombectomy using the Pounce LP Mechanical Thrombectomy device. Under general anesthesia, considering multiple prior operations with fresh surgical incisions in the groin, the right common femoral artery was exposed and accessed from the previous groin incision. Intraoperative angiography confirmed the occlusions (Fig 1, *A* and *B*) and revealed faint reconstitution of the medial plantar artery at the hindfoot through collaterals (Fig 1, *C*). A 7-Fr destination sheath was placed antegrade into the superficial femoral artery and the peroneal artery; the communicating branch of the peroneal artery and distal PT/medial plantar artery were traversed utilizing a 0.035 wire and Navicross catheter (Fig 2, *A*). The wire was exchanged for a 0.014 Hi-Torque Command ES wire (Abbott), and the Pounce LP hydrophilic delivery catheter was advanced into the medial plantar artery over the 0.014 wire. The wire was then exchanged for the dual 4-mm diameter self-expanding nitinol baskets and delivered through the catheter to the medial plantar artery, extending into the midfoot (Fig 2, *B*). The catheter was removed, and the 7 mm × 6.5 cm funnel catheter was moved into position to capture the baskets. The Pounce LP device tracked within the small-caliber vessels smoothly, enabling controlled engagement and removal of thrombus. Two passes achieved successful removal of organized thrombus—predominantly fibrotic, laminated, and lighter in appearance—and subsequent restoration of multiphasic inline flow to the peroneal artery and its communicating branch, which subsequently fed into the distal PT artery and the plantar vessels. Completion angiography confirmed flow to the midfoot through the medial plantar artery (Fig 2, *C*). The patient had a palpable PT pulse at this point. The access site was closed, and the patient was transferred back to the surgical intensive care unit in stable condition.

Following the procedure, a multiphasic PT signal was present at the medial malleolus with distal extension. The patient’s postoperative course was complicated by atrial fibrillation with rapid ventricular response and urinary retention. The incision site remained clean and intact, and the patient was discharged on postoperative day 12 with dry gangrene of the right toe tips and ischemic changes over the dorsal forefoot. On discharge, her foot was warm, with a palpable PT pulse and detectable DP Doppler signal.

**Fig 3:**
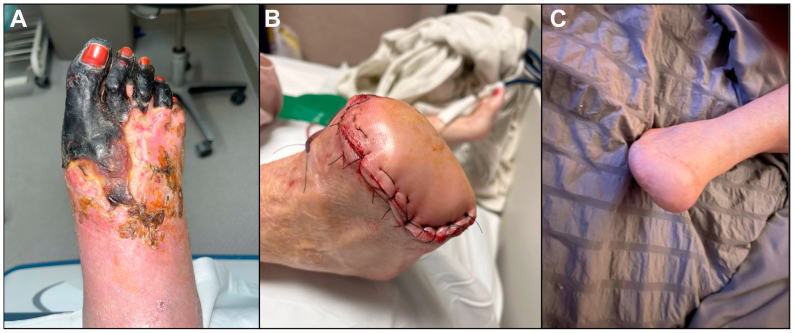
**(A)** Right foot 8 weeks after revascularization using the Pounce LP Thrombectomy System, demonstrating demarcation between forefoot gangrene and healing tissue in the midfoot. **(B)** Transmetatarsal amputation (TMA) performed at 8 weeks after revascularization. **(C)** Fully healed TMA 10 months after the initial presentation to our hospital, achieving limb salvage and ambulatory independence.

Over the ensuring 8 weeks, the distal right forefoot was allowed to demarcate (Fig 3, *A*). The patient then underwent a successful right transmetatarsal amputation (TMA) by podiatry. The midfoot remained well-perfused and viable with no signs of deep or spreading necrosis, achieving limb salvage (Fig 3, *B*). At her 1-week follow-up post-TMA, the patient was pain-free and participating in rehabilitation, and her surgical wound was clean, dry, and healing without signs of infection or dehiscence. Ten months post-TMA, the patient remained independently ambulatory with no recurrence of ischemic symptoms (Fig 3, *C*). She is maintained on therapeutic rivaroxaban for her atrial fibrillation, aspirin, and high-dose statin therapy.

## Discussion

We report the successful use of the Pounce LP Thrombectomy System to achieve revascularization of the pedal vessels and subsequent limb salvage following very distal thromboembolism in an 83-year-old patient with ALI, after multiple failed attempts at both open and percutaneous thromboembolectomy. This case expands the clinical application of the newly introduced, purely mechanical Pounce LP device in 2- to 3-mm vessels beyond its current reported uses and illustrates its feasibility, safety, and effectiveness in the treatment of extremely distal subacute occlusions.

**Fig 4:**
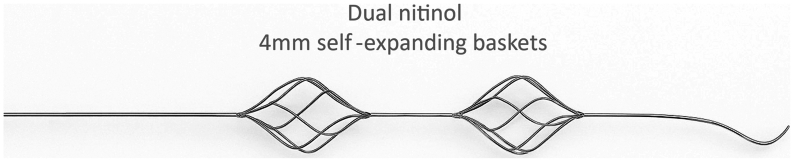
Diagram of the Pounce LP Thrombectomy catheter tip showing the dual nitinol 4-mm self-expanding baskets.

The Pounce LP Thrombectomy System, specifically designed for use in peripheral arteries measuring 2 to 4 mm in diameter, represents a promising advance for patients with severely compromised distal and pedal arterial flow. This fully percutaneous, purely mechanical device utilizes a dual-basket design (Fig 4) to efficiently remove both acute and organized thrombus without the need for thrombolytic agents or aspiration techniques.^8^ Early experience with the Pounce device has demonstrated a procedural success rate of up to 95%, achieving symptom resolution and restoration of pulsatile flow to the foot even in a cohort where nearly 60% of patients presented with subacute or chronic limb ischemia—conditions that often limit the effectiveness of other commonly used thrombectomy devices.^7^ However, its reported use has largely been confined to the femoropopliteal segment, with limited data supporting its application in more distal vessels.^7^ In this case, the Pounce LP Thrombectomy System was successfully utilized to remove organized thrombus from the peroneal artery and its communicating branch to the PT artery and, ultimately, achieve limb salvage after multiple failed open surgical and percutaneous thrombectomy attempts complicated by distal thromboembolism with worsening ischemia.

The Pounce LP device overcomes several limitations associated with the commonly used thrombectomy systems. Mechanical thrombectomy devices such as the JETi Thrombectomy System (Abbott) and Rotarex (Becton Dickinson) have demonstrated efficacy in thrombus removal but are less suitable for small-caliber vessels such as the tibial and pedal arteries, making complete thrombus removal in the distal circulation challenging.^9^ Designed primarily for larger vessels, these devices also carry a higher risk of vessel injury and distal embolization when used in smaller arteries.^9^, ^10^, ^11^ Although aspiration thrombectomy devices such as the Indigo (Penumbra) and AngioJet (Boston Scientific) can be utilized in smaller vessels, they are generally less effective against organized thrombus—more commonly encountered in subacute or recurrent ALI, which was the predominant finding in our case. Thrombus composition is often heterogeneous, and these devices frequently leave behind residual organized clots. In our case, initial use of a popular aspiration thrombectomy device at an outside hospital achieved only partial success by restoring limited flow to the posterior tibial artery before rapid reocclusion and distal embolization occurred. Additionally, the use of these other devices is often associated with the need for adjunctive endovascular procedures, significant blood loss, and systemic complications, including bleeding, renal impairment, and distal embolization.^12^, ^13^, ^14^

Open surgical thromboembolectomy, although traditionally effective, also failed in this case, likely due to the distal location of the thrombus and underlying vessel disease, which limited both the reach and efficacy of a standard balloon thrombectomy device. Collectively, these challenges underscore the need for a dedicated thrombectomy device capable of navigating small-caliber vessels and effectively removing organized thrombus—capabilities that were demonstrated by the Pounce LP Thrombectomy System in this case. Given the subacute nature and distal distribution of the thrombus, the Pounce LP system may have been preferable even at the initial reintervention, offering a more targeted and less invasive strategy than open thromboembolectomy. The Pounce Thrombectomy System Retrospective Registry (PROWL) further supports the efficacy of the Pounce LP device, demonstrating a 97% procedural flow restoration rate in patients with acute, subacute, or chronic limb ischemia, with 82% of subjects not requiring any additional thrombus removal treatments post-Pounce system use.^15^ Although these results are promising, further studies are needed to evaluate long-term outcomes compared with existing devices and technologies. Nonetheless, our experience with this case demonstrates that the Pounce LP Thrombectomy System represents a valuable addition to the endovascular therapy armamentarium.

Although the Pounce LP Thrombectomy System offers notable benefits for treating distal and organized thrombi, certain potential drawbacks associated with the device should be acknowledged. Notably, the device is not an over-the-wire system, which introduces the potential risk of losing wire access with each pass. Although this has not resulted in adverse events in our experience, it can increase procedural complexity and prolong case duration. In cases involving pre-existing stents, the use of the Pounce LP device requires meticulous fluoroscopic guidance due to a theoretical risk of stent displacement or difficulty in device retrieval. Additionally, although the cost is comparable to other similar devices, it remains higher than that of conventional balloon thromboembolectomy. Finally, as with all thrombectomy tools, the risks of distal embolization, vasospasm, and vessel dissection remain present.

## Conclusions

The Pounce LP Thrombectomy System, designed for 2- to 4-mm vessels, was effective in revascularization of distal tibial and pedal arteries, allowing for limb salvage after profound thromboembolism following failed attempts at both open and percutaneous thrombectomy. By addressing the limitations of conventional devices in small-caliber, distal vessels, this technology expands the armamentarium of treatment options for patients with severely compromised arterial flow in the pedal distribution.

## Funding

Research was supported by the Betty Jane and Alfred J. Fisher Vascular Surgery Research Fund.

## Disclosures

K.O. is a speaker/consultant for Surmodics.

## Appendix (online only)

Supplementary Fig Ischemic changes of the right foot upon presentation to our institution.
