# Supplementary material for: Limb salvage using Pounce LP mechanical thrombectomy system after failed open and percutaneous thromboembolectomy
Source: J Vasc Surg Cases Innov Tech. 2025 Jul 29;11(6):101935. doi: 10.1016/j.jvscit.2025.101935 (PMC12445564; doi:10.1016/j.jvscit.2025.101935)

**Supplementary Figure 1.** Ischemic changes of the right foot upon presentation to our institution


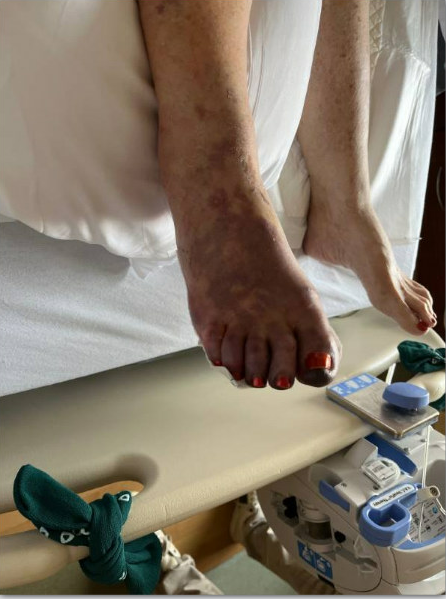

Supplement: Supplementary Fig — Ischemic changes of the right foot upon presentation to our institution. [file mmc1.docx]
